# Implementation of home blood pressure monitoring among French GPs: A long and winding road

**DOI:** 10.1371/journal.pone.0220460

**Published:** 2019-09-11

**Authors:** Giselle Dugelay, Joëlle Kivits, Louise Desse, Jean-Marc Boivin

**Affiliations:** 1 Université de Lorraine, Département de Médecine Générale, Nancy, France; 2 Université de Lorraine, École de Santé Publique, Nancy, France; 3 Université de Lorraine, Apemac, Nancy, France; 4 Centre d’Investigations Clinique Plurithématique 1433 (CIC-P), Inserm, CHRU de Nancy, Nancy, France; Universita degli Studi di Perugia, ITALY

## Abstract

**Background:**

To explore the perception of home blood pressure monitoring (HBPM) by general practitioners (GPs) in everyday practice in order to identify facilitators and barriers to its implementation in daily practice.

**Methods:**

A qualitative study comprising the conduct of six focus groups between October 2016 and February 2017, gathering 41 general practitioners in primary care practice in Lorraine (North Eastern France), with thematic and comprehensive analysis.

**Results:**

The first reasons given by GPs to explain their difficulties with HBPM (Home Blood Pressure Monitoring) implementation were the usual lack of time, material and human resources. However, all of these motives masked other substantial limiting factors including insufficient knowledge regarding HBPM, poor adherence to recommendations on HBPM and fear of losing their medical authority. GPs admitted that HBPM use could enhance patient observance and decrease therapeutic inertia. Despite this observation, most GPs used HBPM only at the time of diagnosis and rarely for follow-up. One explanation for GP reluctance towards HBPM may be, along with guidelines regarding hypertension, HBPM is perceived as being a binding framework and being difficult to implement. This barrier was more predominantly observed among aging GPs than in young GPs and was less frequent when GPs practiced in multidisciplinary health centers because the logistical barrier was no longer present.

**Discussion:**

In order to improve HBPM implementation in everyday practice in France, it is necessary to focus on GP training and patient education. We must also end "medical power" in hypertension management and turn to multidisciplinary care including nurses, pharmacists and patients.

## Introduction

Hypertension is the foremost chronic disease in the world. In 2012, high blood pressure accounted for 30% of individuals over 25 years of age worldwide and a crude increase in the prevalence in the last decade has been observed [1]. In 2017, it accounted for more than 13 million people in France or around 30% of the population over 35 years old [2].

As such, hypertension control is a major public health issue. However, despite recommendations put forward over the past several years to improve its management, hypertension remains insufficiently controlled [3,4]. In Europe, blood pressure (BP) targets are rarely reached, whether patient management is overseen by a general practitioner (GP) or by a specialist [5,6]. In France, 55% of hypertensive patients were adequately controlled in 2015 [7] and only 50.9% in 2017 [2].

A more systematic use of home blood pressure monitoring (HBPM) by GPs is constantly being advanced by learned societies to improve hypertension diagnosis and control[8–10]. In a great majority of cases, OBP (Office Blood Pressure) measurement is not reliable [11]. In comparison, HBPM is often associated with good hypertensive management, as Verberk and al. already pointed out in 2007 [12–14]. Indeed, the detection of white coat and masked hypertension [15] as well patient compliance improvement [16,17], owing to HBPM, allows better BP control and in addition to having a good better prognostic impact [18,19]. It is moreover an inexpensive tool, easy to implement by GPs, and its use has been both well tolerated by patients [20] and democratized throughout the world [21,22]. However, HBPM use remains insufficient in many countries [23,24], particularly in France [25,26].

HBPM protocols and GP acceptance of the HBPM method differ from one country to another. In France, the HBPM protocol consists in measuring BP three times, spaced by a few minutes, in the morning and three times in the evening before bedtime, during three successive days. It is recommended to confirm the diagnosis of high blood pressure with an out-of-office BP measurement. In Japan, it is recommended to measure BP, twice per occasion, within one hour of waking-up, before breakfast, and in the evening before retiring, after one to two minutes rest. The measurement period should be as long as possible [27]. In Europe, it is recommended to measure BP at home twice in the morning and twice in the evening for seven days [3].

In 2009, Boivin et al. observed that HBPM use by French general practitioners increased from 70% in 2004 to 92% in 2009. However, only 21% recommended HBPM in the majority of their patients, while 71% were only casual users. Their study revealed that adherence by French general practitioners to hypertension guidelines remained insufficient, particularly with regard to an under-use of HBPM, as well as a lack of knowledge of its diagnostic and prognostic value. [26] In 2015, a qualitative study led in Netherlands highlighted that GPs implemented out-of-office BP monitoring but showed a strong preference for ambulatory blood pressure monitoring (ABPM) even when there was poor tolerance of the method. [28] In 2017, Kronish et al. conducted a semi quantitative study (based on the nominal group) which enabled similar barriers to our study to be highlighted but not intimate reasons, such as the fear of losing medical power. [29]

In quantitative studies, reasons given by GPs for HBPM under-use remain systematically the same in many countries: lack of time, logistical difficulties and lack of knowledge or confidence in hypertension guidelines [30–33]. However, previousthese studies did not really explore the main reasons explaining the lack of HBPM appropriation by GPs. Most of the published studies, in France, were quantitative studies using closes-ended questionnaires that did not allow the GPs to express themselves freely.

In the light of the above, the aim of the present study was to explore, through a qualitative study, general practitioners’ perception of HBPM use in current practice in order to identify existing factors levering and limiting its systematic implementation in daily practice.

## Material and method

This study was conducted in accordance with COREQ criteria (Consolidated Criteria for Reporting Qualitative research) [34].

### Procedure

This qualitative study was carried out in the form of six focus groups, conducted among 41 general practitioners in the eastern region of France. The GPs were recruited from phone list and mailing list provided by the Faculty of Medicine, through the Council of the Medical Association and by knowledge network. This method of qualitative data collection was selected for its ability to produce, within a short time-frame, a wide variety of ideas, opinions and beliefs currently perceived on a given topic [20–27]. An interview guide was developed beforehand to discuss various topics on self-measurement (Table 1) (GD, LD, JK).

**Table 1:** Interview guide: Advantages and brakes linked to HPBM.

***Physician related***:
▪ What does HBPM represent for you?
▪ How does HBPM impact your practice? Positively and negatively?
▪ Why have you decided to integrate HBPM into your practice?
▪ What are your sources of information regarding HBPM?
▪ How is HBPM perceived by the specialists with whom you collaborate?
***Equipment-related***:
▪ Do you advise your patients regarding the choice of equipment?
▪ How do you ensure access to HBPM to your patients?
▪ What advice do you give regarding data collection?
***Patient-related***:
▪ How does HBPM impact your patients? Positively and negatively?
▪Are there some patients to whom you do not propose HBPM?
▪ Do you have fears regarding HBPM use by your patients?
▪ Do you generally trust the results brought by your patients?
***Logistic-related***:
▪ How do you manage your time when using HBPM?
▪ Do you encounter any organizational/logistical difficulty in HBPM implementation?
***Economics***:
▪ Do you think that this practice can lower healthcare costs?
▪ Does HBPM cause an additional cost for physicians and healthcare system?

Each meetingErreur de traductionFEach meeting was recorded and subsequently fully transcribed to gather all statements. Each focus group was composed of GPs using HBPM more or less regularly. Erreur de traductionFocus groups were led by a moderator and an observer (GD, LD). The observer’s role was to collect non-verbal behavior, noting silences and hesitations. The role of the moderator was to ensure that each of the GPs could speak on each theme. It was not a questionnaire but a semi-structured open discussion. The duration of the focus groups was about two hours. We ensured for each group that all of the themes in the interview guide had been addressed. There were reminders when the moderator estimated that the number of physicians answering was insufficient.

At the end of each meeting, a short questionnaire was distributed to collect socio-demographic data. Study data collection was conducted from October 2016 to February 2017. Data collection was performed until data saturation.

### Recruitment

Sampling was carried out among a population of general practitioners working in a primary care office setting. Variability in age, gender, type and place of practice were taken into consideration for constituting the different GP panels. The first group comprised teachers and GPs trained in the use of HBPM (some GPs had a university degree in hypertension and cardiovascular risk). The second group comprised GPs occasionaly working with one of the authors and known to them as non-user.

### Data analysis

All participating GPs gave their agreement to the recording of the interviews. All of the comments expressed during each focus group were meticulously transcribed, preserving the anonymity of those involved. All participants were informed regarding the goal of this study, the modalities of focus group attainment and the processing of collected data.

All of the statements were translated by the investigators. A thematic and comprehensive analysis was performed and allowed the elaboration of an analysis grid according to the following major themes: inventory of HBPM use; facilitators and barriers related to HBPM use; logistical strategies. Analysis of the data was performed by three researchers (GD, LD, JK).

## Results

Forty-one (41) GPs participated in the focus groups. The characteristics of the studied population are summarized in Table 2, with a synthesis of the key points described in Table 3.

**Table 2:** Characteristics of participating GPs.

Physician characteristics	FG 1 (1)	FG 2	FG 3	FG 4	FG 5	FG 6	Total
Gender							
Female	2	3	1	2	2	5	15
Male	6	4	6	6	2	2	26
Age categories							
< 40	2	2	1	0	1	2	8
[40–60[	4	3	5	3	3	4	22
60 and over	2	2	1	5	0	1	11
Practice area							
Rural	2	0	4	1	0	0	7
Semi-rural	4	1	1	4	0	3	13
Urban	2	6	2	3	4	4	21
Mode of practice							
Individually	4	1	2	5	2	2	16
Collectively (2)	4	6	5	3	2	5	25
Number of devices (3)							
0	1	0	0	0	1	3	5
1	0	3	3	4	0	4	14
[2 à 5]	5	4	4	4	3	0	20
≥5	2	0	0	0	0	0	2
Loaning of the device provided by the healthcare system? (4)							
yes	8	7	7	8	4	2	36
no	0	0	0	0	0	5	5

^(1)^ medical practice with only one doctor

^(2)^ medical practice is composed of several GPs

^(3)^ number of blood pressure monitors available from the general practitioner for a loan for his patients

^(4)^ a blood pressure device was made available to each GP by the French health insurance and was intended for HBPM by patients

**Table 3:** Perceived limits and benefits of HBPM.

Limiting factors	Levering factors
**HBPM use brakes in GPs daily practice**: Lack of knowledge of some GPs: rules of HBPM use, blood pressure target, masked hypertension, validated devices list Lack of confidence in the method: still questioning the diagnostic value of HBPM compared with OBP	**HBPM benefits in GPs daily practice**: A tool against GPs’ therapeutic inertia **(raised by a few GPs)** Reduction in antihypertensive drug prescription and drug side effects
**HBPM misuse by the patients**: Inadequate or poor measurement conditions Abusive and obsessive use Transcription errors	**HBPM benefits for patients (raised by a few GPs)**: Better patient involvement in his/her health and better therapeutic compliance
**Insufficient logistical resources (raised by the majority)**: Lack of human resources when working alone Underuse of medical support and websites dedicated to HBPM	**Sufficient logistical resources (raised by a few GPs)**: Sufficient human resources when practicing in group (nurse, secretary …)
**Lack of material resources (raised by the majority)**: Number of loaned devices still insufficient for a regular use Frequent use of non validated devices	**Enough material resources (raised by a few GPs)**: Devices loaned by GPs, pharmacist, patient family members… Purchase by the patients (affordable cost of the devices)
**A threat for medical authority**: Impossible to give a justification to medical inertia The patients becomes an actor of his medical management	**Decrease in health care cost**: Better blood pressure control and better prognostic value Reduction in specialized examinations and consultations

### Barriers related to HBPM implementation

#### Lack of confidence in the HBPM method

One of the major barriers identified in this study was the lack of confidence of GPs in the HBPM method, generating a great variability in GP practices. GPs mentioned transcription errors and wrong measurement conditions. They referred, for example, to an unfulfilled rest time, an obsessive use by the patient, or BP measurement during anxiety or pain.

A proposal for HBPM was made when GPs suspected “white coat hypertension”. However, they did not always trust HBPM results when the latter were unexpected, such as in the case for masked hypertension. Most of the time, they only used OBP, which they judged sufficiently accurate, to follow up hypertension, and were still adjusting treatments without confirmation from an out-of-office BP measurement (that is to say either an ABPM or an HBPM).

*“There must have been (white coat hypertension)* …*yes definitely*… *but afterwards*, *if we measure several times before”*

(Man 43Y FG6)

*“I do not completely trust electronic devices*, *even those in hospitals”*

(Man 57Y FG3)

#### Availability of blood pressure devices

Numerous sources of access to devices were mentioned. However, GPs still considered the number of loaned devices as a factor limiting HBPM practice and several physicians said they would use HBPM more frequently if they had more loaned devices.

To compensate for the lack of device, some GPs tended to ask their patients to buy their own BP device. However, this solution was not consensual as other GPs considered that patients should not own a device because of the risk of obsessive use. GPs also described an economic barrier and unequal access for patients who cannot always afford to buy a BP device.

*“To have a device at home*, *I think that it is not always a good thing (talking about obsessive use)”*

(Man 37Y FG1)

“It’s a real access barrier in my area… clearly there are a lot of patients who don’t buy the device because they can’t afford it”

(Man 37Y FG1)

It was therefore often still necessary for the GPs to lend a BP device to their patient, nevertheless few of them make the effort to invest in a pool of loaned devices. Moreover, when discussing the choice of device, only a few physicians in our panel knew the lists of validated BP devices.

“There must be a list of recommended devices? I suppose so”

(Woman 31Y FG5)

#### Insufficient logistical resources

Among all criticisms made regarding HBPM, the lack of time and the lack of human resources emerged as major barriers for HBPM implementation.

Time spent educating patients and to analyzing results was perceived as a genuine limitation to HBPM implementation.

“Patient education may not be the fifth reason for consultation in a quarter of an hour”

(Man 35Y FG3) (in a protesting tone)

“I often have to do the average”

(Man 55Y FG1) (In a disappointed tone)

*“Who really does the average of all the measurements*? *(*…*) I do a rough estimation”*

(Man 33Y FG6)

In particular, GPs complained that HBPM requires human resources and a certain level of organization which are not available within the French health-care system. Indeed, delegation of tasks is not always available in the French system’s organization.

*“Why not*, *but in this case*, *the health care system will have to train and pay qualified nurses*…*why don*’*t they come to our office*!*”*

(Woman 50Y FG3)

#### From knowing to trusting scientific guidelines

From the discussions, we noted deficiencies in GP knowledge regarding hypertension management. They did not always know when, how and how often an HBPM should be performed. Moreover, GPs were often unfamiliar with BP objectives. While OBP thresholds were more commonly known, ambulatory BP thresholds were most often inaccurate. Lastly, few physicians were aware of new hypertension concepts such as isolated ambulatory hypertension, and were therefore unable to recognize this type of hypertension when faced with the latter.

*“If BP are beyond 130*/*85mmHg with HBPM*, *it*’*s too high*!*”*

(Man 62Y FG4)

Even when guidelines regarding hypertension were known, GPs, and particularly the older ones, had some reluctance in tightly applying these guidelines.

*“In medicine*, *in the younger generation*, *only recommendations matter! Sometimes*, *the younger generation of doctors confuses recommendations with an obligation”*

*(Man*, *47Y FG3)*

They did not always acknowledge scientific evidence regarding HBPM efficiency on BP control. Another reason for poor BP control was that GPs did not adhere to guidelines regarding BP targets. In fact, they often underestimated cardiovascular consequences due to a moderately elevated BP. These findings were more prevalent within the older generation of GPs.

*“In real life*, *in terms of public health*: *does “half a cmHg”*, *or so*, *really make a difference in patients*’ *lives*?*”*

(Man 54 Y FG1) (In a protesting tone)

*“We do not treat in order to have good BP measures*, *we treat to protect against CVD (cardiovascular disease) risk”*

(Man 59Y FG3)

*“If hypertension has no impact on the cardiologist*’*s consultation* … *I am less rigorous for the BP target”*

(Man 59Y FG3)

It is worth highlighting that when it comes to medical practice in multidisciplinary health centers (particularly in rural and semi-rural areas with young practitioners), HBPM use seemed to be more frequent and closer to the recommendations because the logistical barrier was no longer present.

*“We are organized in a multidisciplinary health center with the pharmacists*. *So we prescribe a loan of blood pressure device and the pharmacies lend the device to the patient and explain the modalities of HBPM”*

(Man 29Y FG1)

#### HBPM, a threat to medical authority

Most GPs, and particularly the older generation of GPs, expressed their concerns regarding patient empowerment when practicing HBPM. The main issue was the risk of self-adaptation of treatment by the patient.

“It can make them change their treatment”

(Woman 63Y FG1)

*“They (the patients) are controlled in self measurement and they tell you “you know*, *it*’*s been a month since I have stopped taking the treatment and you see*, *it is still good”*

(Woman 63Y FG6)

“*Some patients say that there is no need to put this medication on the prescription because they don*’*t take it anymore”* (because the BP is controlled in HBPM)

(Woman 32Y FG6)

By measuring their BP, patients become actors of their hypertension management and are therefore empowering themselves. This last aspect seemed to particularly worry GPs.

*“(About not giving BP thresholds to the patient)*: *I tell a patient that he has to write down the numbers on the device and that there is no reason to try to predict what I will think about it”*

(Woman 55Y FG1)

*“You should not explain too much because*, *if you do*, *you lose power over people”*

(Man 52Y FG2)

*“Do they (patients) really need to know*…?*”*

(Woman 50Y FG4)

For example, some GPs did not tell patients their “real” OBP values in order to not have to manage the patient’s reactions. In the same way, some GPs did not tell the patients their BP target in order not to have to justify their therapeutic inertia. Indeed, this “wait and see” attitude of some GPs faced with an elevated BP could no longer be justified to a patient who was educated on the subject and involved in HBPM.

*“Well*, *you give him a bogus BP value*! *Why do you measure BP again*?*”*

(Woman 50Y FG4)

*“There*, *with an electronic device you cannot cheat* … *patients will see BP values”*

(Man 62Y FG4)

Notwithstanding the above, the expression of risk regarding their medical authority and power did not prevent them from quoting advantages for HBPM implementation, notably better patient compliance.

### Advantages related to HBPM implementation

Most GPs agreed to say that HBPM could enhance patient compliance since it enhanced patient comprehension of hypertension and its consequences. Furthermore, GPs highlighted that HBPM use was linked to a decrease in antihypertensive drug side effects and thus increased patient therapeutic compliance.

*“It has been proven that empowering them*, *involving them in their health was beneficial”*

(Man 55Y FG1)

GPs sometimes recognized the implication of therapeutic inertia. In the same way, they agreed to say that HBPM practice was a valuable tool against this. Indeed, one cause of physician inertia was due to a lack of confidence in their OBP in some cases, such as white coat hypertension.

*“One determinant of therapeutic inertia is*: *‘You are nervous today*… *So*, *this time*, *we will ignore your BP measures*’. *Now*, *patients come with HBPM that were taken at rest*! *So*, *we can no longer hide behind a white coat effect when we do not really want to change the treatment*!*”*

(Man 55Y FG1)

## Discussion

This study highlighted a great reluctance of GPs regarding a regular use of HBPM. Whereas using HBPM when making a diagnosis of hypertension for the first time was rather well accepted, they conversely expressed true reservations with regard to the systematic use of HBPM, in other words before every reassessment of antihypertensive treatment.

Our qualitative study enabled exploring certain barriers which were not clearly explained by previous French studies. The studies on the appropriation of HBPM entitled Megamet [26] did not allow, any more than FLASH studies [2] to explore real obstacles to HBPM use. These studies were conducted using closed-ended questions that did not allow the GPs’ feelings to be explored. This qualitative approach has been implemented in other countries but not in France [20,29,35,36]. The focus group approach of the study allowed GPs to freely express themselves without the restrictive nature of a closed-questions survey. Indeed, the given pretext of lacking time would often disguise the true reasons highlighted by our qualitative study.

Regardless of the country and despite the differences in health care systems, whether it be in the United Kingdom, Germany or France, GPs have all put forward logistical difficulties and lack of time to explain their lack of HBPM use in daily practice. [26,37,38]. In addition to the above, other barriers for HBPM implementation emerged from our analysis. A lack of scientific knowledge and awareness of hypertension guidelines were noted in the present study, as also described in other studies [39]. Furthermore, some studies have shown that GPs often suggested much more frequent home measurements than recommended and that their interpretation of HBPM results were often suboptimal [40]. Other studies quoted that even when physicians were aware of guidelines, there was still a gap between knowledge and implementation of the recommendations due to a lack of adherence to guidelines. [41] The lack of confidence in the HBPM method has also been noted in other countries, including Japan. In a Japanese questionnaire survey, some physicians still answered that OBP was more reliable than HBPM [42].

In Europe, it was the Nice 2011 guidelines that introduced, for the first time, the obligation to carry out ambulatory measurements of BP to confirm the permanence of hypertension [43]. In 2013, the ESH working group on BP monitoring has proposed a number of recommendations for HBPM. The 2010 Canadian Hypertension Education Program recommendation also included HBPM in the diagnosis of hypertension. [44]

In France, the definition of hypertension is still based on clinical measurements[45], even though, since 2011, it is recommended to confirm hypertension with HBPM before the start of antihypertensive drug therapy [4]. Previous French guidelines on hypertension rely exclusively on OBP. Indeed, hypertension was defined by SBP ≥ 140mmHg and/or a DBP ≥ 90mmHg, measured in a medical office and confirmed by at least two OBP measures of three successive consultations during a three to six months period. [45,46]

This is maybe one of the reasons why some GPs, and especially those from the older generation, may feel it difficult to step back from previous norms and tell their patients that OBP is no longer accurate at all and that, now, it is HBPM that is far more reliable for diagnosis and follow-up of hypertension [47].

In the present study, GPs rarely made a connection between under-use of HBPM and poor BP control. GPs still remain convinced that poor BP control is solely due to patients, without ever mentioning their share of responsibility in terms of therapeutic inertia-which is rarely admitted. Therapeutic inertia has been defined as the failure of health-care provider to initiate or intensify therapy when therapeutic goals are not reached. In a study conducted in 2010, French GPs attributed poor BP control to poor patient compliance [48]. Therapeutic inertia was not spontaneously mentioned whereas the latter is one of the most important causes of poor BP control worldwide and particularly in France [49–51]. Indeed, in a French study conducted in 2007, therapeutic inertia was estimated at 85%. Comparatively, in United States, therapeutic inertia was estimated at 63% [51]. Furthermore, even if GPs judge HBPM as a good tool to improve patient observance and decrease therapeutic inertia, these benefits don’t seem to be sufficient to make them use HBPM more frequently [35,52].

GPs, and especially older GPs, also expressed their fear regarding patient empowerment, especially with regard to a loss of medical authority. In France, as in other countries, GPs are reluctant to tightly implement recommendations, which they associate as an attack on their medical power[53,54]. Most GPs base their medical practice on their individual experiences and on an intuitive approach [55]. With the HBPM method, patients are educated and “know some of the rules” of the management of their hypertension. Hence, if GPs do not “apply the rule”, they will have to explain the reasons for not doing so to their patients.

Some of the GPs interviewed were rather paternalistic. The reasons for this paternalism may have been that the pre-requirement for a shared medical decision is therapeutic information and education[56]. Therapeutic education requires time and resources they often do not have (as a reminder, the GPs interviewed were mostly installed alone or in areas of low medical density). GPs therefore do not give themselves the time or the means to form a shared medical decision and decide on their own what is good for their patients [57]. This paternalistic aspect did not appear in a similar British study. The British healthcare system allows a multidisciplinary care involving the doctor, the nurse and a health care assistant (HCA). The GPs in this study delegated some of the BP management and this may explain this less paternalistic aspect [58].

In addition, our study revealed that certain GPs do not always disclose BP measurements if they are too high. GPs stated that they did not want to worry patients regarding high BP values, but in fact it would appear that they do not want to take time to explain HBPM and to argue with patients. GPs would like to avoid confronting themselves with a binding framework [43,44]. If patients are educated and involved in the management of their hypertension as a result of HBPM practice, GPs will be forced to follow recommendations.

Finally, several barriers need to be overcome in order to spread HBPM use, namely the need to improve GPs’ scientific knowledge, fight against their medical beliefs, and accept a form of patient empowerment [59]. An area for improvement could be to simplify the schedule for self-measurement, and/or to have sufficient flexibility to allow adaptation to individual routine, so that, in the long term the motivation of GPs and patients will be not impacted [30,36,58].

However, this ongoing reluctance by GPs towards the HBPM method could lead to the conclusion that implementation of HBPM is not going to improve, regardless of the efforts made by the health care system. In such case, an alternative to HBPM could be the office blood pressure measurement, whose validity is now well established [60,61]. Indeed, in the Canadian guidelines, it is now recommended to evaluate BP with an unattended OBP measurement as performed in the SPRINT study. In this latter study, the adjustment of antihypertensive drug was based on a mean of three BP measurements at an office visit, while the patient was seated after five minutes of quiet rest, and with an automated measurement system [62]. However, the current French medical office organization does not always allow this new method of BP assessment, since it requires that the patient remains alone in a private room.

The strengths of the present study are a triangulation in several stages of our research: a triangulation during data collection and analysis and a triangulation of investigators. Its design in the form of focus group, in semi-structured interviews, furthermore allowed a group dynamic to be created, obtaining a rich debate. Limitations of this study were partially linked to the methodology. Indeed, focus group constitution is dependent on GP availability or GP motivation and, as such, can generate a selection bias. Finally, the focus group method induces a desirability bias, albeit less significant than with individual interviews.

## Conclusion and perspectives

GPs express a lack of confidence in the HBPM method, and a lack of confidence in scientific guidelines. Although they have partial knowledge of them, they deplore insufficient logistical resources, and express a fear of losing medical authority by regular use of HBPM. On the other hand, they acknowledge that HBPM improves patient compliance, decreases antihypertensive drug side effects and decreases therapeutic inertia.

The time when physicians were the only actors in hypertension management is over. It seems essential to put an end to medical power, reflecting a practice of medicine in which physicians’ knowledge is uniquely prominent and orientates therapeutic choices, excluding patients’ points of view and lay knowledge. Our study shows how it is now necessary to encompass a health-care approach shared by all, i.e. physicians, nurses, pharmacists and patients, and accept the evolution of medical practice towards a doctor-patient therapeutic alliance.

Several scientific experiments in which the patient shared a healthcare role with their nurse and/or pharmacist have shown good outcomes [63–65].

Similar programs should be launched and expanded to all hypertensive patients. In order to allow for this to occur, all hypertensive patients should be educated regarding HBPM by their GPs, or by a care network in which access to a BP device should be developed. For instance, why not reimburse BP devices for all hypertensive patients, or set up a booking system in conjunction with pharmacies or a care network? A healthcare program focused on multidisciplinary care, as implemented in Canada with the Canadian Hypertension Education Program (CHEP), could contribute to a more systematic use of HBPM and thus to a better BP control.

Another means to spread HBPM use should also include teaching this method to GPs in the early years of their medical training. GPs should now realize that HBPM is an essential tool for BP management, and that OBP is something of the past, as it is too inaccurate. GPs, especially older GPs, will have to step back from previous recommendations regarding hypertension, which stated that several office BP measurements were sufficient to make a diagnosis of hypertension.

Changing GPs habits regarding hypertension, especially in the older GP generation, will certainly be a long and winding road.
